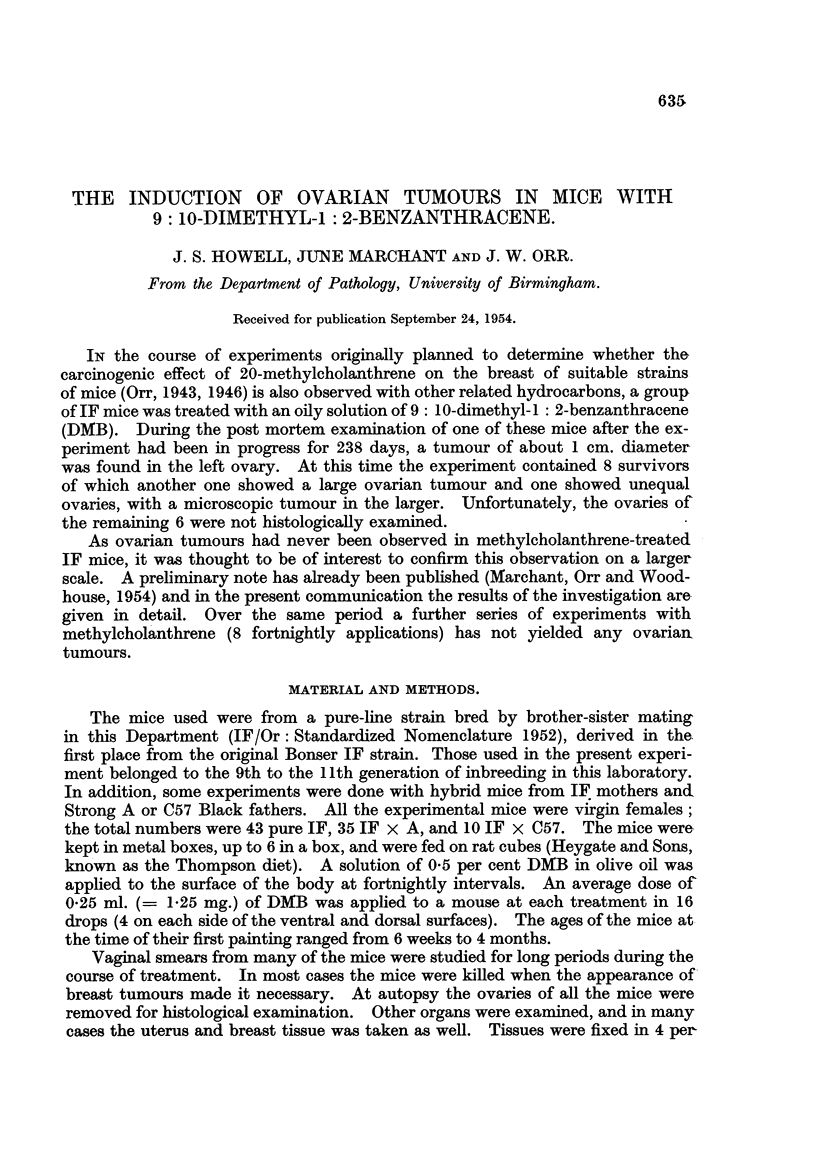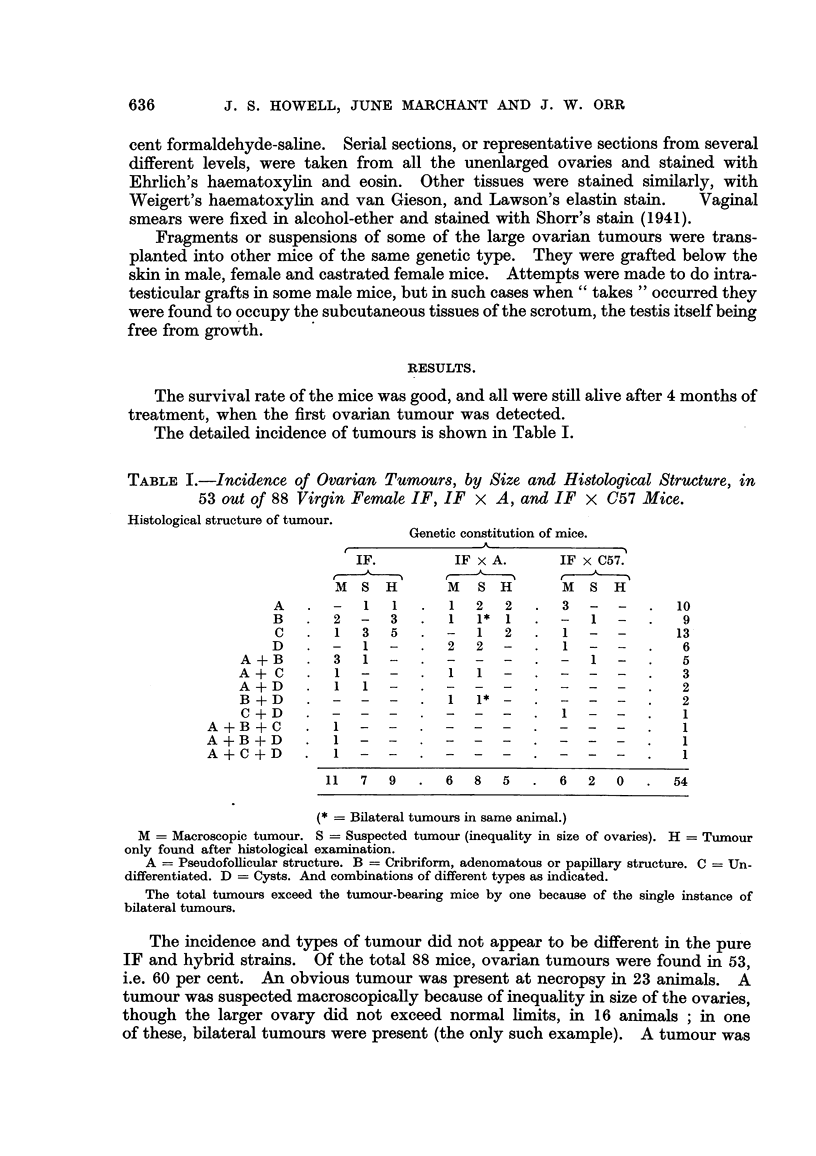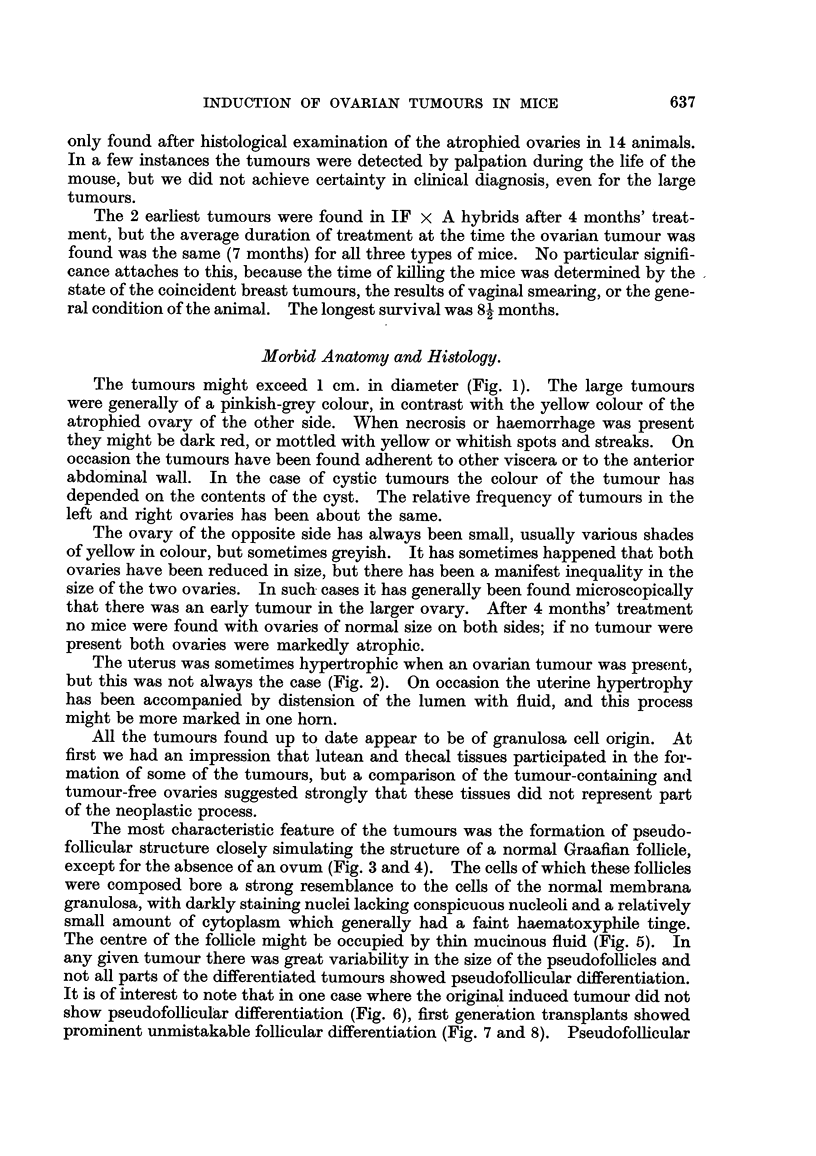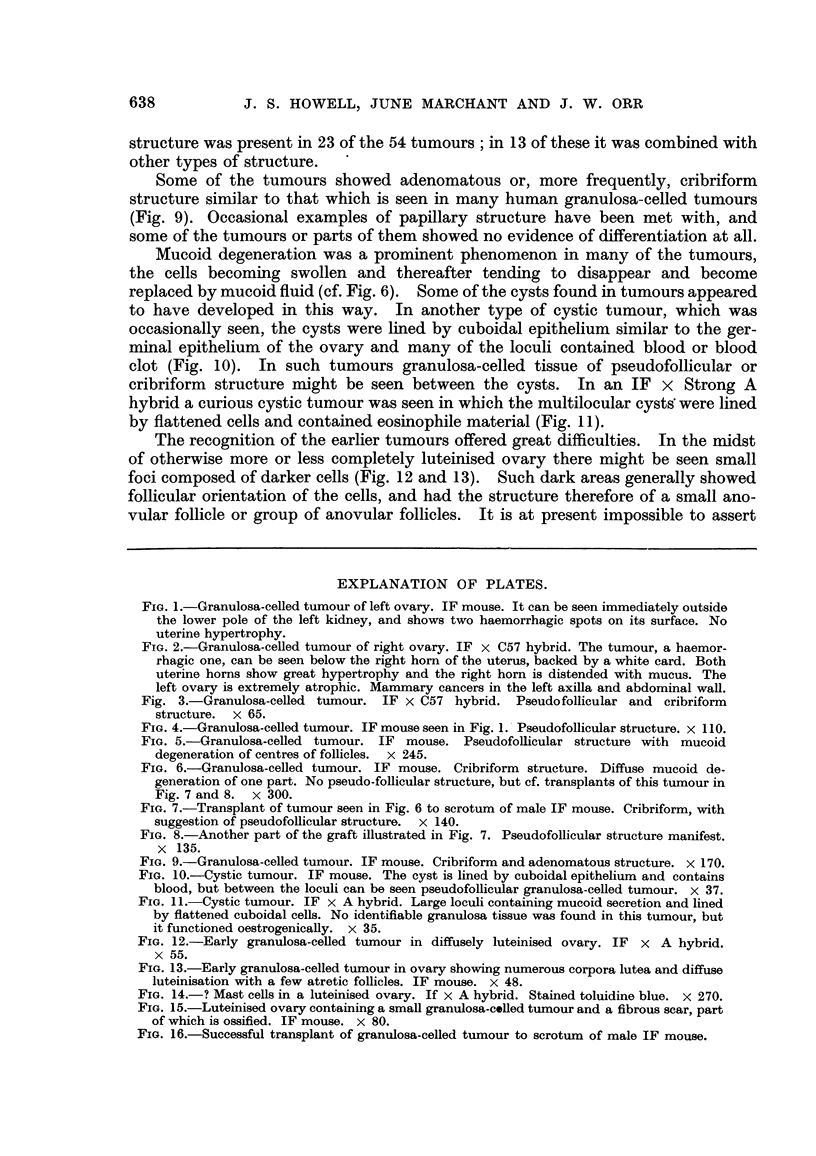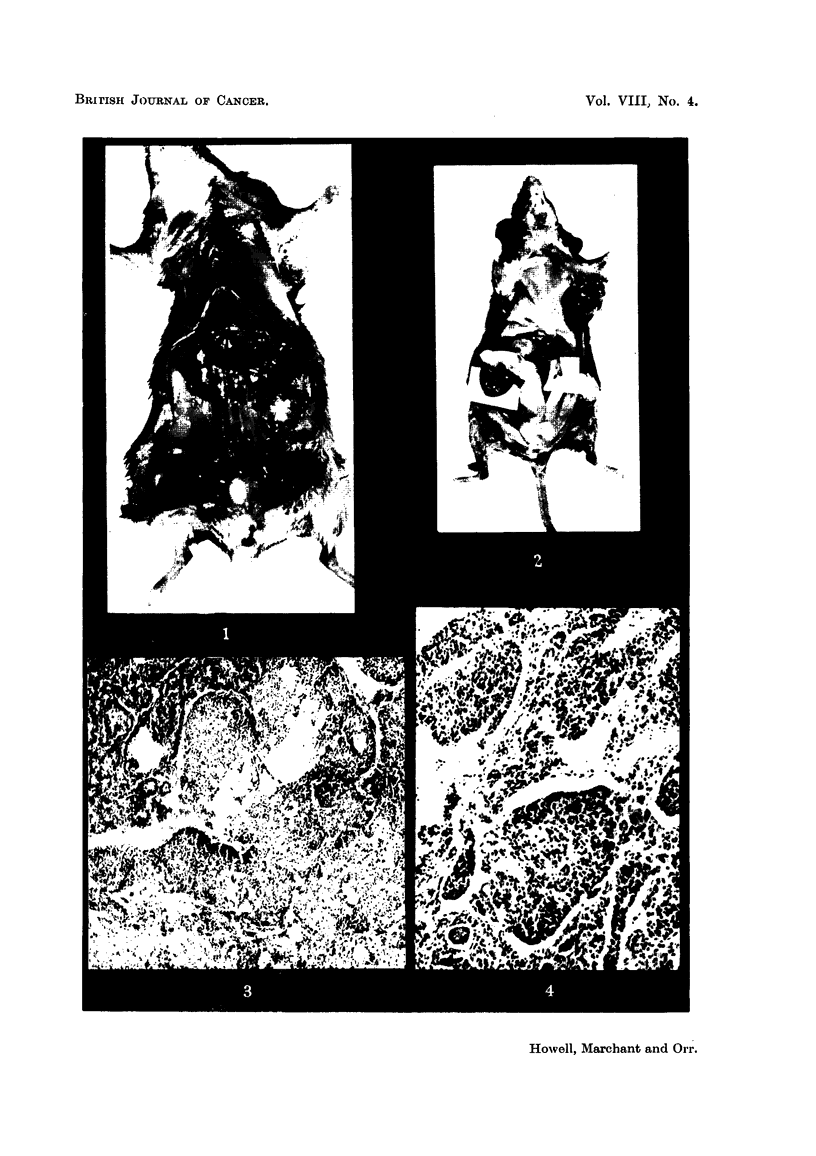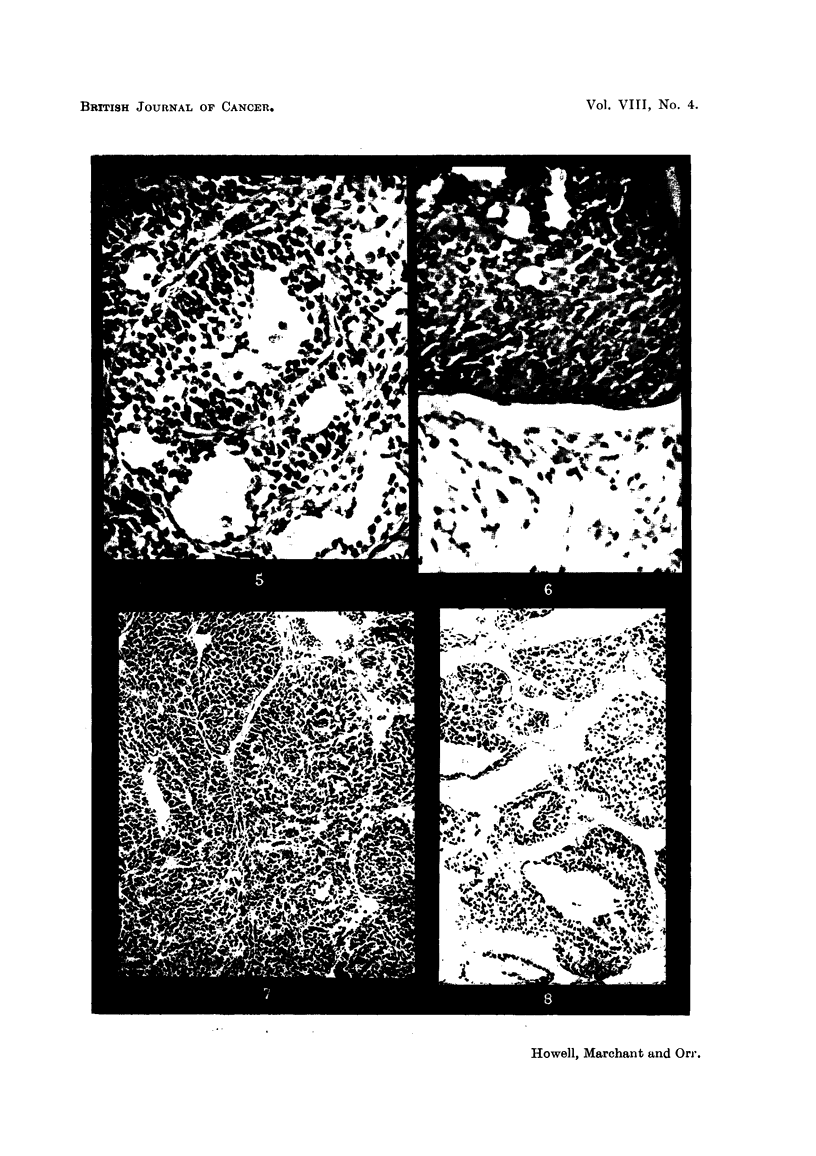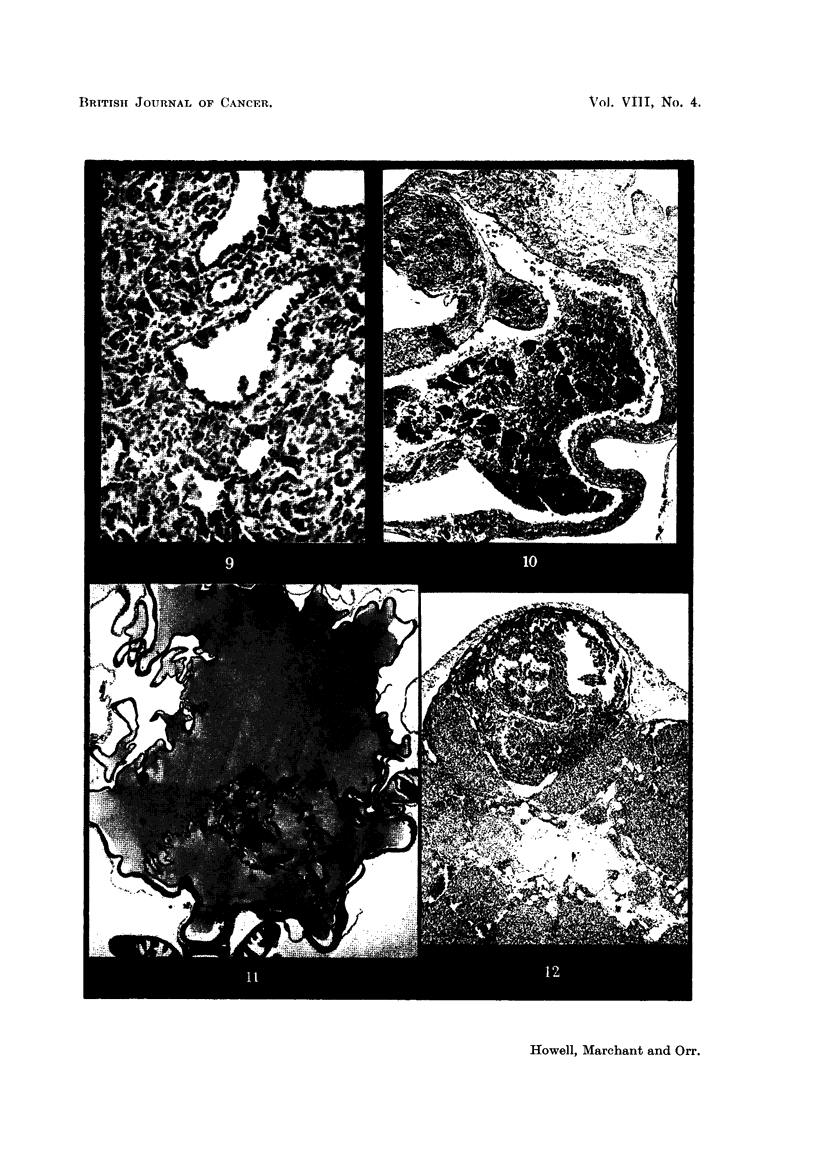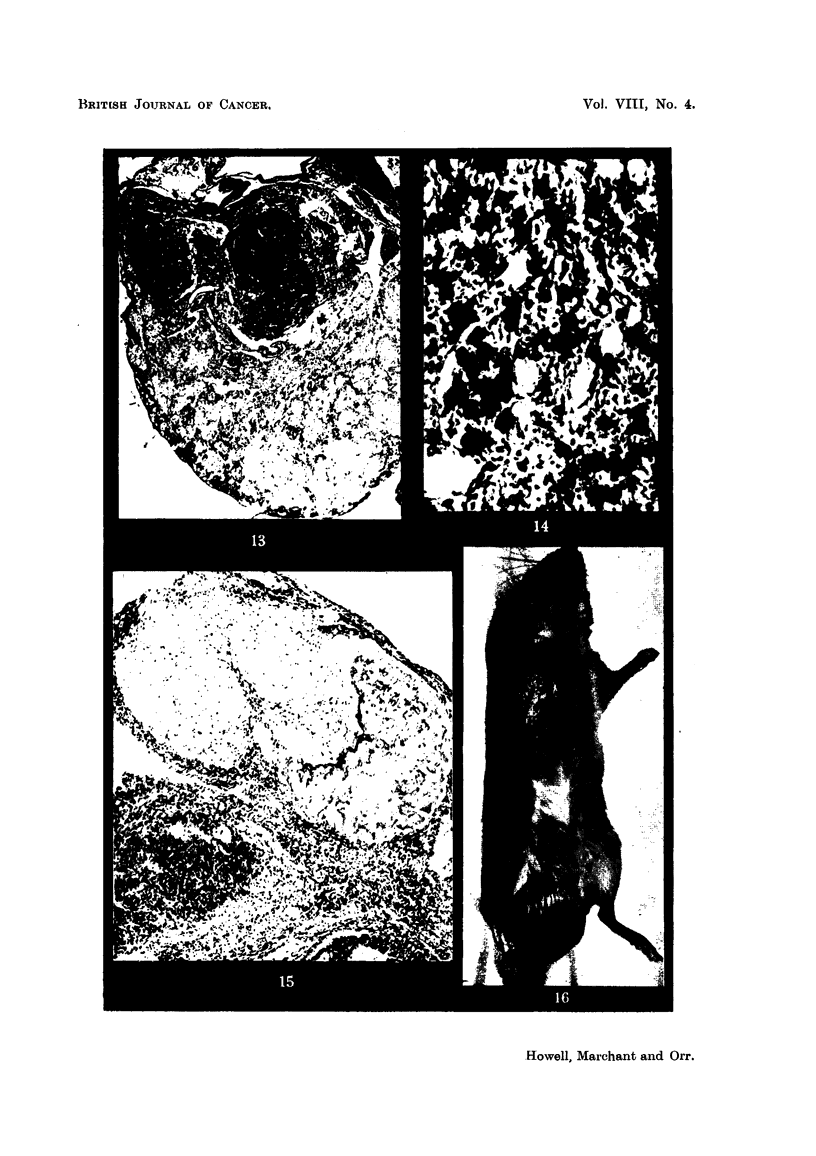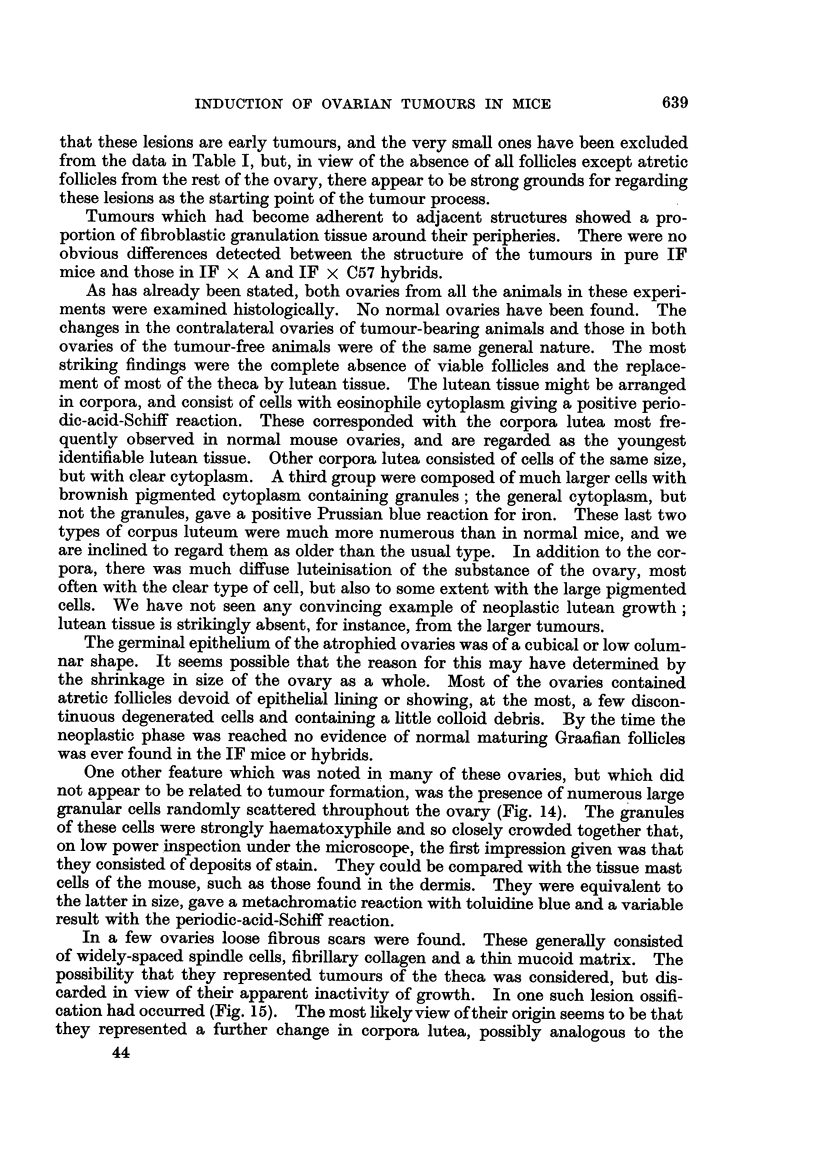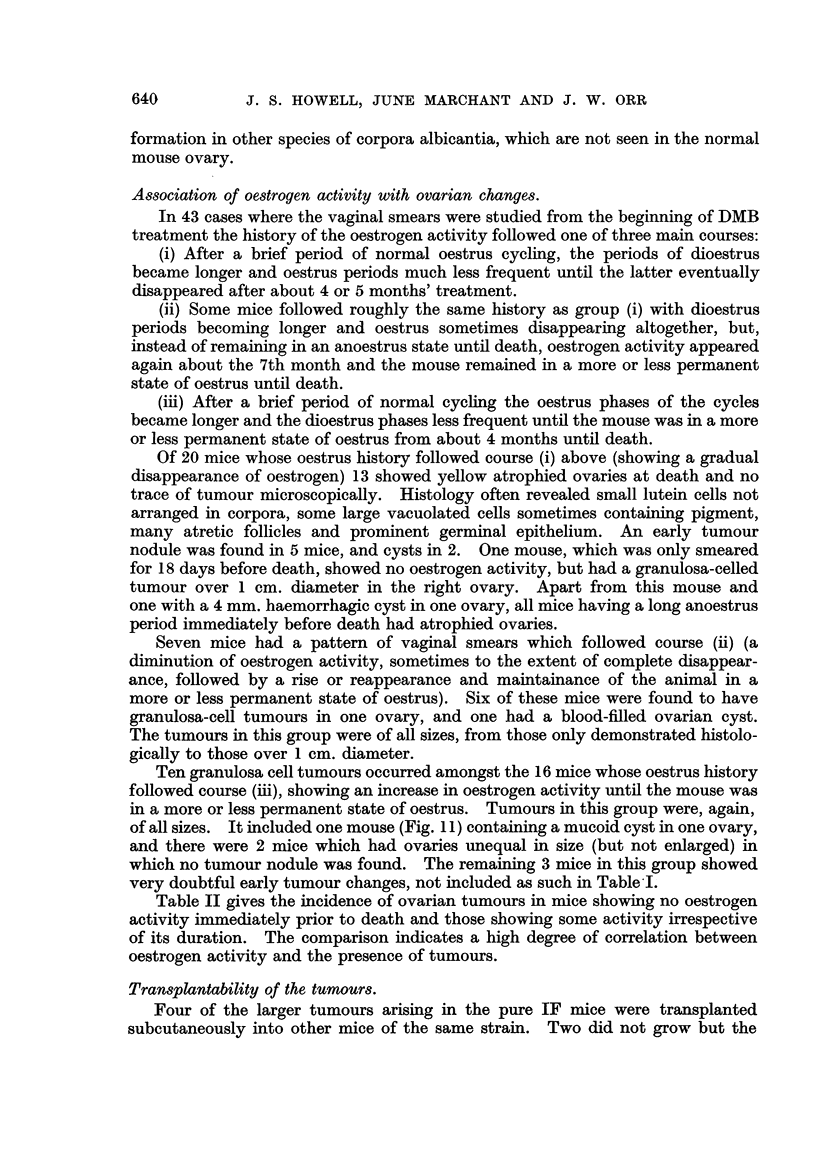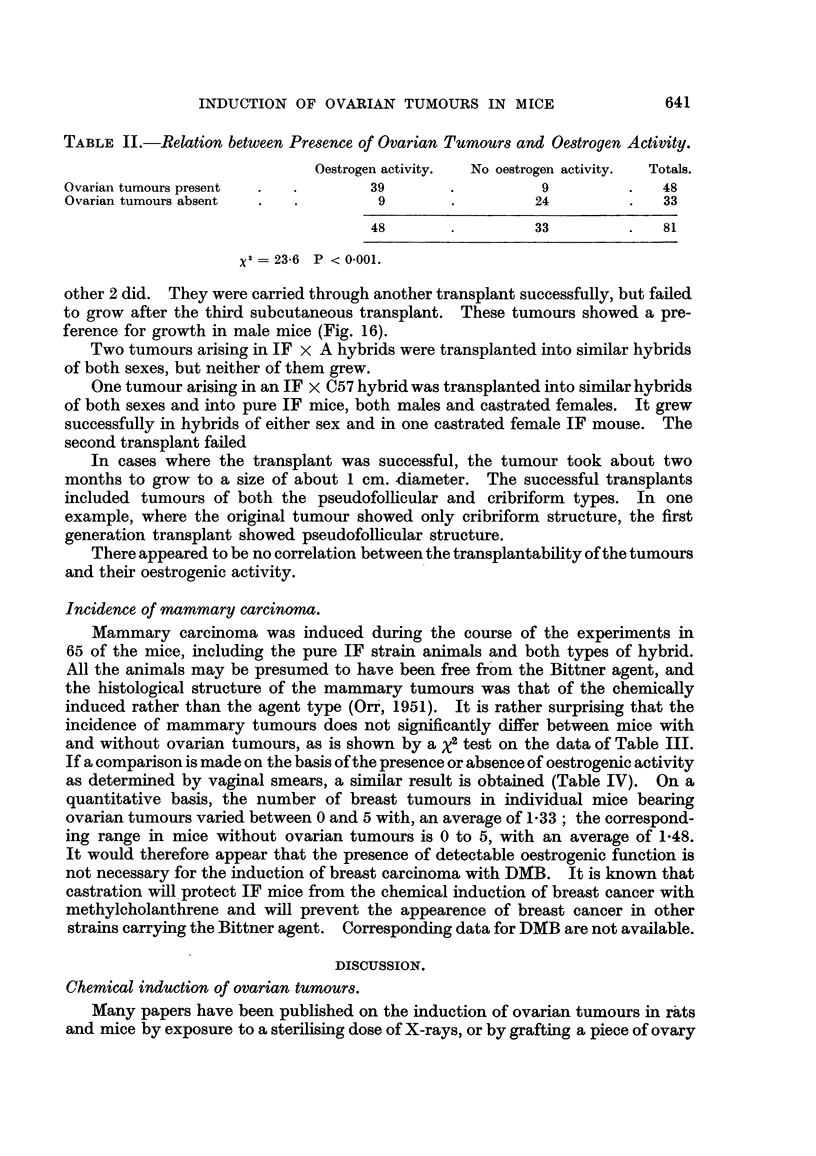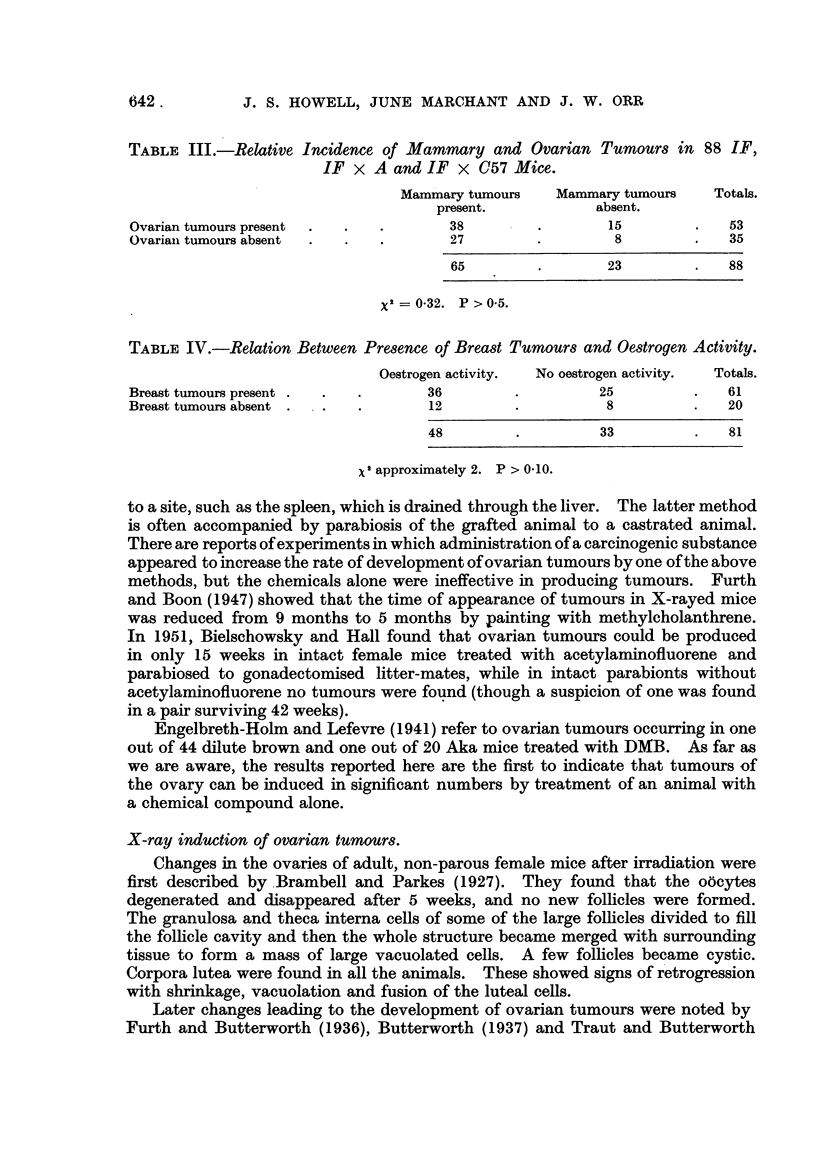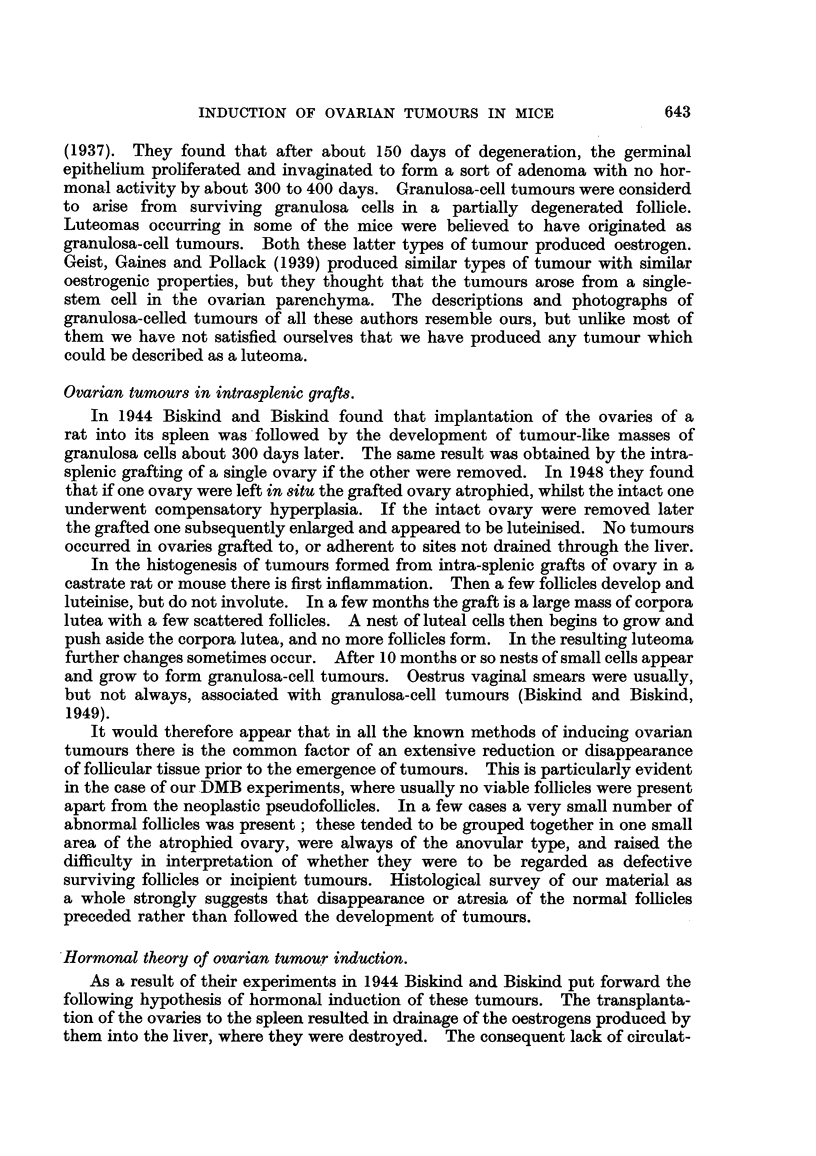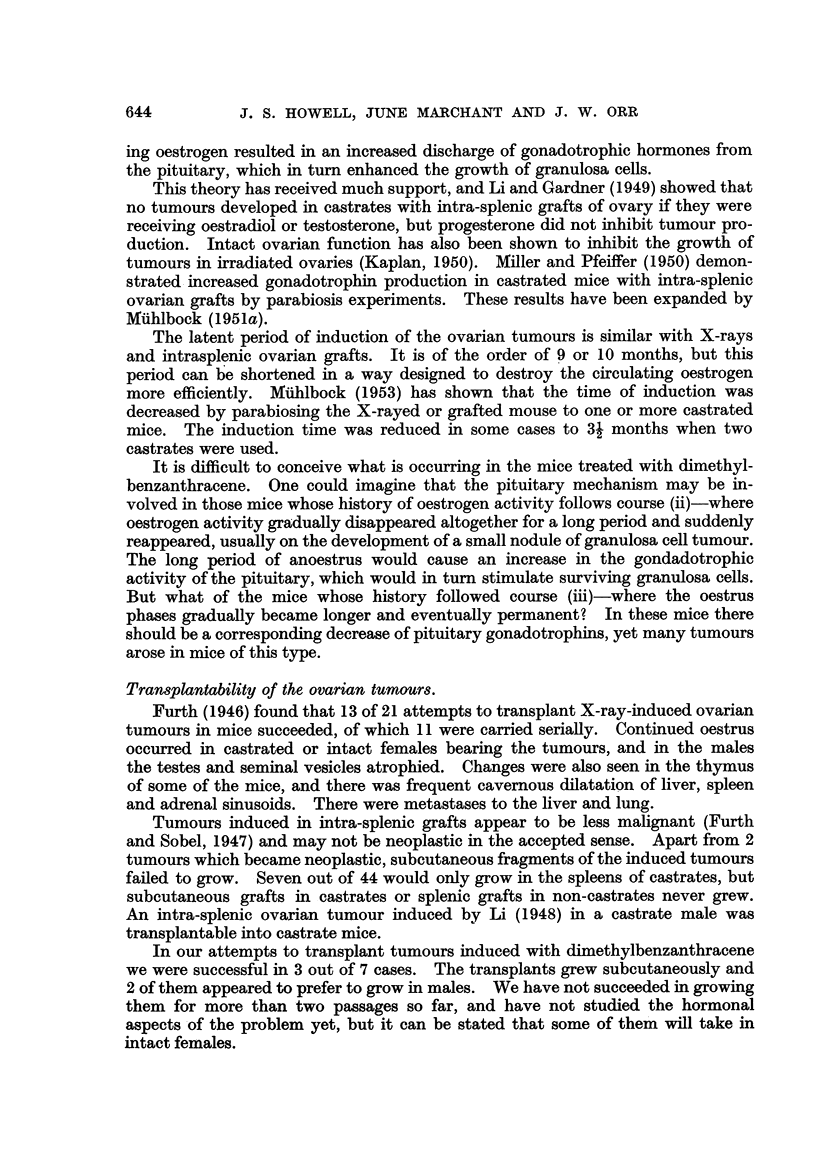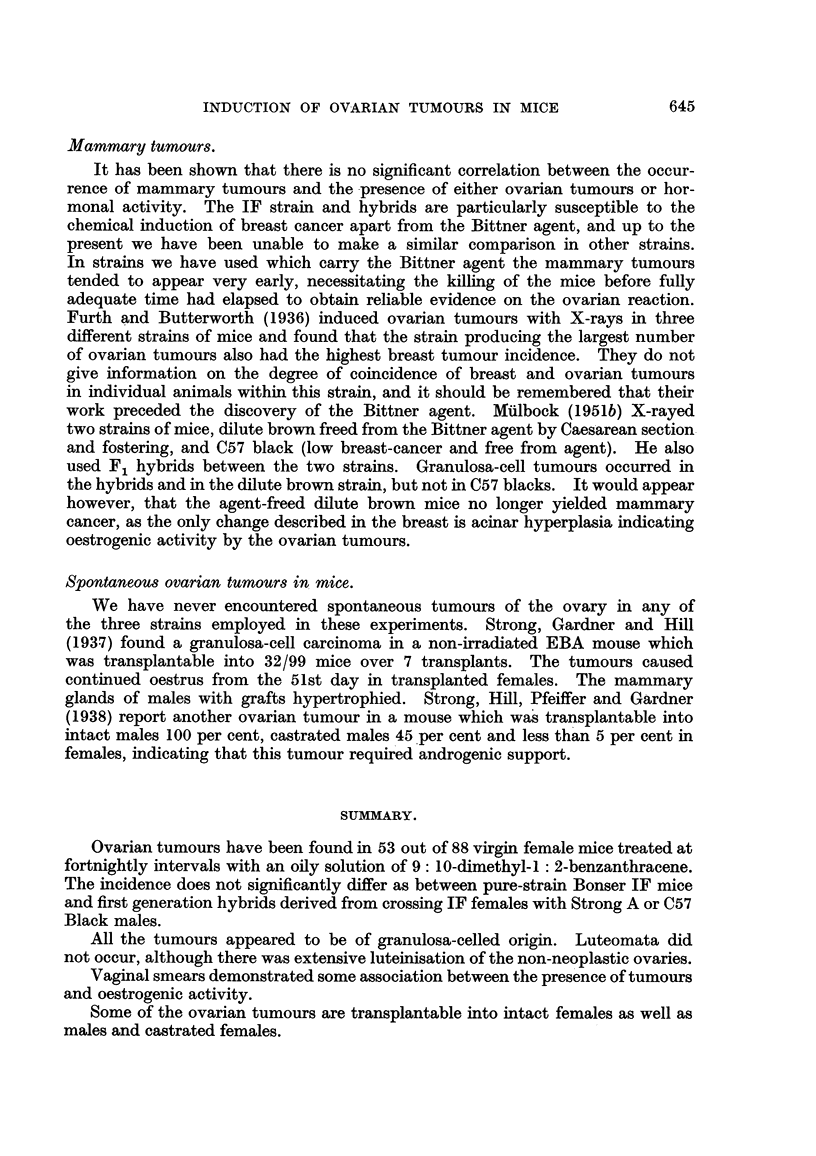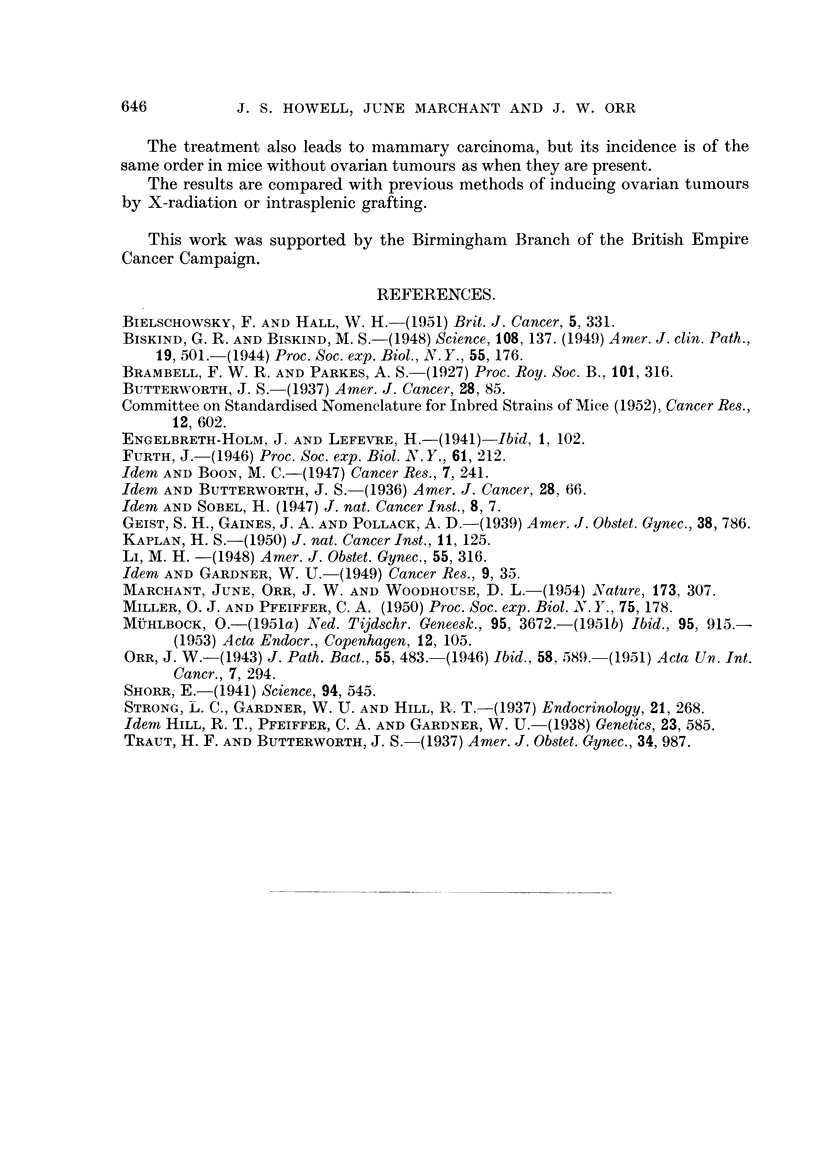# The Induction of Ovarian Tumours in Mice with 9: 10-Dimethyl-1: 2-Benzanthracene

**DOI:** 10.1038/bjc.1954.69

**Published:** 1954-12

**Authors:** J. S. Howell, June Marchant, J. W. Orr

## Abstract

**Images:**


### THE INDUCTION OF OVARIAN TUMOURS IN MICE WITH 9 : 10-DIMETHYL-1 : 2-BENZANTHRACENE.

J. S. HOWELL, JUNE MARCHANT AND J. W. ORR.

*From the Department of Pathology, University of Birmingham.*

Received for publication September 24, 1954.

IN the course of experiments originally planned to determine whether the carcinogenic effect of 20-methylcholanthrene on the breast of suitable strains of mice (Orr, 1943, 1946) is also observed with other related hydrocarbons, a group of IF mice was treated with an oily solution of 9 : 10-dimethyl-1 : 2-benzanthracene (DMB). During the post mortem examination of one of these mice after the experiment had been in progress for 238 days, a tumour of about 1 cm. diameter was found in the left ovary. At this time the experiment contained 8 survivors of which another one showed a large ovarian tumour and one showed unequal ovaries, with a microscopic tumour in the larger. Unfortunately, the ovaries of the remaining 6 were not histologically examined.

As ovarian tumours had never been observed in methylcholanthrene-treated IF mice, it was thought to be of interest to confirm this observation on a larger scale. A preliminary note has already been published (Marchant, Orr and Woodhouse, 1954) and in the present communication the results of the investigation are given in detail. Over the same period a further series of experiments with methylcholanthrene (8 fortnightly applications) has not yielded any ovarian tumours.

### MATERIAL AND METHODS.

The mice used were from a pure-line strain bred by brother-sister mating in this Department (IF/Or : Standardized Nomenclature 1952), derived in the first place from the original Bonser IF strain. Those used in the present experiment belonged to the 9th to the 11th generation of inbreeding in this laboratory. In addition, some experiments were done with hybrid mice from IF mothers and Strong A or C57 Black fathers. All the experimental mice were virgin females ; the total numbers were 43 pure IF, 35 IF × A, and 10 IF × C57. The mice were kept in metal boxes, up to 6 in a box, and were fed on rat cubes (Heygate and Sons, known as the Thompson diet). A solution of 0·5 per cent DMB in olive oil was applied to the surface of the body at fortnightly intervals. An average dose of 0·25 ml. (= 1·25 mg.) of DMB was applied to a mouse at each treatment in 16 drops (4 on each side of the ventral and dorsal surfaces). The ages of the mice at the time of their first painting ranged from 6 weeks to 4 months.

Vaginal smears from many of the mice were studied for long periods during the course of treatment. In most cases the mice were killed when the appearance of breast tumours made it necessary. At autopsy the ovaries of all the mice were removed for histological examination. Other organs were examined, and in many cases the uterus and breast tissue was taken as well. Tissues were fixed in 4 per cent formaldehyde-saline. Serial sections, or representative sections from several different levels, were taken from all the unenlarged ovaries and stained with Ehrlich's haematoxylin and eosin. Other tissues were stained similarly, with Weigert's haematoxylin and van Gieson, and Lawson's elastin stain. Vaginal smears were fixed in alcohol-ether and stained with Shorr's stain (1941).

Fragments or suspensions of some of the large ovarian tumours were transplanted into other mice of the same genetic type. They were grafted below the skin in male, female and castrated female mice. Attempts were made to do intratesticular grafts in some male mice, but in such cases when " takes " occurred they were found to occupy the subcutaneous tissues of the scrotum, the testis itself being free from growth.

### RESULTS.

The survival rate of the mice was good, and all were still alive after 4 months of treatment, when the first ovarian tumour was detected.

The detailed incidence of tumours is shown in Table I.

TABLE I.—*Incidence of Ovarian Tumours, by Size and Histological Structure, in 53 out of 88 Virgin Female IF, IF × A, and IF × C57 Mice.*

| Histological structure of tumour. | Genetic constitution of mice. | | | | | | | | | |
|---|---|---|---|---|---|---|---|---|---|---|
| | IF. | | | IF × A. | | | IF × C57. | | | |
| | M | S | H | M | S | H | M | S | H | |
| A | – | 1 | 1 | 1 | 2 | 2 | 3 | – | – | 10 |
| B | 2 | – | 3 | 1 | 1* | 1 | – | 1 | – | 9 |
| C | 1 | 3 | 5 | – | 1 | 2 | 1 | – | – | 13 |
| D | – | 1 | – | 2 | 2 | – | 1 | – | – | 6 |
| A + B | 3 | 1 | – | – | – | – | – | 1 | – | 5 |
| A + C | 1 | – | – | 1 | 1 | – | – | – | – | 3 |
| A + D | 1 | 1 | – | – | – | – | – | – | – | 2 |
| B + D | – | – | – | 1 | 1* | – | – | – | – | 2 |
| C + D | – | – | – | – | – | – | 1 | – | – | 1 |
| A + B + C | 1 | – | – | – | – | – | – | – | – | 1 |
| A + B + D | 1 | – | – | – | – | – | – | – | – | 1 |
| A + C + D | 1 | – | – | – | – | – | – | – | – | 1 |
| | 11 | 7 | 9 | 6 | 8 | 5 | 6 | 2 | 0 | 54 |

(* = Bilateral tumours in same animal.)

M = Macroscopic tumour. S = Suspected tumour (inequality in size of ovaries). H = Tumour only found after histological examination.

A = Pseudofollicular structure. B = Cribriform, adenomatous or papillary structure. C = Undifferentiated. D = Cysts. And combinations of different types as indicated.

The total tumours exceed the tumour-bearing mice by one because of the single instance of bilateral tumours.

The incidence and types of tumour did not appear to be different in the pure IF and hybrid strains. Of the total 88 mice, ovarian tumours were found in 53, i.e. 60 per cent. An obvious tumour was present at necropsy in 23 animals. A tumour was suspected macroscopically because of inequality in size of the ovaries, though the larger ovary did not exceed normal limits, in 16 animals ; in one of these, bilateral tumours were present (the only such example). A tumour was only found after histological examination of the atrophied ovaries in 14 animals. In a few instances the tumours were detected by palpation during the life of the mouse, but we did not achieve certainty in clinical diagnosis, even for the large tumours.

The 2 earliest tumours were found in IF × A hybrids after 4 months' treatment, but the average duration of treatment at the time the ovarian tumour was found was the same (7 months) for all three types of mice. No particular significance attaches to this, because the time of killing the mice was determined by the state of the coincident breast tumours, the results of vaginal smearing, or the general condition of the animal. The longest survival was $8\frac{1}{2}$ months.

*Morbid Anatomy and Histology.*

The tumours might exceed 1 cm. in diameter (Fig. 1). The large tumours were generally of a pinkish-grey colour, in contrast with the yellow colour of the atrophied ovary of the other side. When necrosis or haemorrhage was present they might be dark red, or mottled with yellow or whitish spots and streaks. On occasion the tumours have been found adherent to other viscera or to the anterior abdominal wall. In the case of cystic tumours the colour of the tumour has depended on the contents of the cyst. The relative frequency of tumours in the left and right ovaries has been about the same.

The ovary of the opposite side has always been small, usually various shades of yellow in colour, but sometimes greyish. It has sometimes happened that both ovaries have been reduced in size, but there has been a manifest inequality in the size of the two ovaries. In such cases it has generally been found microscopically that there was an early tumour in the larger ovary. After 4 months' treatment no mice were found with ovaries of normal size on both sides; if no tumour were present both ovaries were markedly atrophic.

The uterus was sometimes hypertrophic when an ovarian tumour was present, but this was not always the case (Fig. 2). On occasion the uterine hypertrophy has been accompanied by distension of the lumen with fluid, and this process might be more marked in one horn.

All the tumours found up to date appear to be of granulosa cell origin. At first we had an impression that lutean and thecal tissues participated in the formation of some of the tumours, but a comparison of the tumour-containing and tumour-free ovaries suggested strongly that these tissues did not represent part of the neoplastic process.

The most characteristic feature of the tumours was the formation of pseudofollicular structure closely simulating the structure of a normal Graafian follicle, except for the absence of an ovum (Fig. 3 and 4). The cells of which these follicles were composed bore a strong resemblance to the cells of the normal membrana granulosa, with darkly staining nuclei lacking conspicuous nucleoli and a relatively small amount of cytoplasm which generally had a faint haematoxyphile tinge. The centre of the follicle might be occupied by thin mucinous fluid (Fig. 5). In any given tumour there was great variability in the size of the pseudofollicles and not all parts of the differentiated tumours showed pseudofollicular differentiation. It is of interest to note that in one case where the original induced tumour did not show pseudofollicular differentiation (Fig. 6), first generation transplants showed prominent unmistakable follicular differentiation (Fig. 7 and 8). Pseudofollicular structure was present in 23 of the 54 tumours ; in 13 of these it was combined with other types of structure.

Some of the tumours showed adenomatous or, more frequently, cribriform structure similar to that which is seen in many human granulosa-celled tumours (Fig. 9). Occasional examples of papillary structure have been met with, and some of the tumours or parts of them showed no evidence of differentiation at all.

Mucoid degeneration was a prominent phenomenon in many of the tumours, the cells becoming swollen and thereafter tending to disappear and become replaced by mucoid fluid (cf. Fig. 6). Some of the cysts found in tumours appeared to have developed in this way. In another type of cystic tumour, which was occasionally seen, the cysts were lined by cuboidal epithelium similar to the germinal epithelium of the ovary and many of the loculi contained blood or blood clot (Fig. 10). In such tumours granulosa-celled tissue of pseudofollicular or cribriform structure might be seen between the cysts. In an IF × Strong A hybrid a curious cystic tumour was seen in which the multilocular cysts were lined by flattened cells and contained eosinophile material (Fig. 11).

The recognition of the earlier tumours offered great difficulties. In the midst of otherwise more or less completely luteinised ovary there might be seen small foci composed of darker cells (Fig. 12 and 13). Such dark areas generally showed follicular orientation of the cells, and had the structure therefore of a small anovular follicle or group of anovular follicles. It is at present impossible to assert

---

EXPLANATION OF PLATES.

FIG. 1.—Granulosa-celled tumour of left ovary. IF mouse. It can be seen immediately outside the lower pole of the left kidney, and shows two haemorrhagic spots on its surface. No uterine hypertrophy.

FIG. 2.—Granulosa-celled tumour of right ovary. IF × C57 hybrid. The tumour, a haemorrhagic one, can be seen below the right horn of the uterus, backed by a white card. Both uterine horns show great hypertrophy and the right horn is distended with mucus. The left ovary is extremely atrophic. Mammary cancers in the left axilla and abdominal wall.

Fig. 3.—Granulosa-celled tumour. IF × C57 hybrid. Pseudofollicular and cribriform structure. × 65.

FIG. 4.—Granulosa-celled tumour. IF mouse seen in Fig. 1. Pseudofollicular structure. × 110.

FIG. 5.—Granulosa-celled tumour. IF mouse. Pseudofollicular structure with mucoid degeneration of centres of follicles. × 245.

FIG. 6.—Granulosa-celled tumour. IF mouse. Cribriform structure. Diffuse mucoid degeneration of one part. No pseudo-follicular structure, but cf. transplants of this tumour in Fig. 7 and 8. × 300.

FIG. 7.—Transplant of tumour seen in Fig. 6 to scrotum of male IF mouse. Cribriform, with suggestion of pseudofollicular structure. × 140.

FIG. 8.—Another part of the graft illustrated in Fig. 7. Pseudofollicular structure manifest. × 135.

FIG. 9.—Granulosa-celled tumour. IF mouse. Cribriform and adenomatous structure. × 170.

FIG. 10.—Cystic tumour. IF mouse. The cyst is lined by cuboidal epithelium and contains blood, but between the loculi can be seen pseudofollicular granulosa-celled tumour. × 37.

FIG. 11.—Cystic tumour. IF × A hybrid. Large loculi containing mucoid secretion and lined by flattened cuboidal cells. No identifiable granulosa tissue was found in this tumour, but it functioned oestrogenically. × 35.

FIG. 12.—Early granulosa-celled tumour in diffusely luteinised ovary. IF × A hybrid. × 55.

FIG. 13.—Early granulosa-celled tumour in ovary showing numerous corpora lutea and diffuse luteinisation with a few atretic follicles. IF mouse. × 48.

FIG. 14.—? Mast cells in a luteinised ovary. If × A hybrid. Stained toluidine blue. × 270.

FIG. 15.—Luteinised ovary containing a small granulosa-celled tumour and a fibrous scar, part of which is ossified. IF mouse. × 80.

FIG. 16.—Successful transplant of granulosa-celled tumour to scrotum of male IF mouse.

1

2

3

4

Howell, Marchant and Orr.

Howell, Marchant and Orr.

9

10

11

12

Howell, Marchant and Orr.

Howell, Marchant and Orr.

that these lesions are early tumours, and the very small ones have been excluded from the data in Table I, but, in view of the absence of all follicles except atretic follicles from the rest of the ovary, there appear to be strong grounds for regarding these lesions as the starting point of the tumour process.

Tumours which had become adherent to adjacent structures showed a proportion of fibroblastic granulation tissue around their peripheries. There were no obvious differences detected between the structure of the tumours in pure IF mice and those in IF × A and IF × C57 hybrids.

As has already been stated, both ovaries from all the animals in these experiments were examined histologically. No normal ovaries have been found. The changes in the contralateral ovaries of tumour-bearing animals and those in both ovaries of the tumour-free animals were of the same general nature. The most striking findings were the complete absence of viable follicles and the replacement of most of the theca by lutean tissue. The lutean tissue might be arranged in corpora, and consist of cells with eosinophile cytoplasm giving a positive periodic-acid-Schiff reaction. These corresponded with the corpora lutea most frequently observed in normal mouse ovaries, and are regarded as the youngest identifiable lutean tissue. Other corpora lutea consisted of cells of the same size, but with clear cytoplasm. A third group were composed of much larger cells with brownish pigmented cytoplasm containing granules ; the general cytoplasm, but not the granules, gave a positive Prussian blue reaction for iron. These last two types of corpus luteum were much more numerous than in normal mice, and we are inclined to regard them as older than the usual type. In addition to the corpora, there was much diffuse luteinisation of the substance of the ovary, most often with the clear type of cell, but also to some extent with the large pigmented cells. We have not seen any convincing example of neoplastic lutean growth ; lutean tissue is strikingly absent, for instance, from the larger tumours.

The germinal epithelium of the atrophied ovaries was of a cubical or low columnar shape. It seems possible that the reason for this may have determined by the shrinkage in size of the ovary as a whole. Most of the ovaries contained atretic follicles devoid of epithelial lining or showing, at the most, a few discontinuous degenerated cells and containing a little colloid debris. By the time the neoplastic phase was reached no evidence of normal maturing Graafian follicles was ever found in the IF mice or hybrids.

One other feature which was noted in many of these ovaries, but which did not appear to be related to tumour formation, was the presence of numerous large granular cells randomly scattered throuphout the ovary (Fig. 14). The granules of these cells were strongly haematoxyphile and so closely crowded together that, on low power inspection under the microscope, the first impression given was that they consisted of deposits of stain. They could be compared with the tissue mast cells of the mouse, such as those found in the dermis. They were equivalent to the latter in size, gave a metachromatic reaction with toluidine blue and a variable result with the periodic-acid-Schiff reaction.

In a few ovaries loose fibrous scars were found. These generally consisted of widely-spaced spindle cells, fibrillary collagen and a thin mucoid matrix. The possibility that they represented tumours of the theca was considered, but discarded in view of their apparent inactivity of growth. In one such lesion ossification had occurred (Fig. 15). The most likely view of their origin seems to be that they represented a further change in corpora lutea, possibly analogous to the formation in other species of corpora albicantia, which are not seen in the normal mouse ovary.

*Association of oestrogen activity with ovarian changes.*

In 43 cases where the vaginal smears were studied from the beginning of DMB treatment the history of the oestrogen activity followed one of three main courses:

(i) After a brief period of normal oestrus cycling, the periods of dioestrus became longer and oestrus periods much less frequent until the latter eventually disappeared after about 4 or 5 months' treatment.

(ii) Some mice followed roughly the same history as group (i) with dioestrus periods becoming longer and oestrus sometimes disappearing altogether, but, instead of remaining in an anoestrus state until death, oestrogen activity appeared again about the 7th month and the mouse remained in a more or less permanent state of oestrus until death.

(iii) After a brief period of normal cycling the oestrus phases of the cycles became longer and the dioestrus phases less frequent until the mouse was in a more or less permanent state of oestrus from about 4 months until death.

Of 20 mice whose oestrus history followed course (i) above (showing a gradual disappearance of oestrogen) 13 showed yellow atrophied ovaries at death and no trace of tumour microscopically. Histology often revealed small lutein cells not arranged in corpora, some large vacuolated cells sometimes containing pigment, many atretic follicles and prominent germinal epithelium. An early tumour nodule was found in 5 mice, and cysts in 2. One mouse, which was only smeared for 18 days before death, showed no oestrogen activity, but had a granulosa-celled tumour over 1 cm. diameter in the right ovary. Apart from this mouse and one with a 4 mm. haemorrhagic cyst in one ovary, all mice having a long anoestrus period immediately before death had atrophied ovaries.

Seven mice had a pattern of vaginal smears which followed course (ii) (a diminution of oestrogen activity, sometimes to the extent of complete disappearance, followed by a rise or reappearance and maintainance of the animal in a more or less permanent state of oestrus). Six of these mice were found to have granulosa-cell tumours in one ovary, and one had a blood-filled ovarian cyst. The tumours in this group were of all sizes, from those only demonstrated histologically to those over 1 cm. diameter.

Ten granulosa cell tumours occurred amongst the 16 mice whose oestrus history followed course (iii), showing an increase in oestrogen activity until the mouse was in a more or less permanent state of oestrus. Tumours in this group were, again, of all sizes. It included one mouse (Fig. 11) containing a mucoid cyst in one ovary, and there were 2 mice which had ovaries unequal in size (but not enlarged) in which no tumour nodule was found. The remaining 3 mice in this group showed very doubtful early tumour changes, not included as such in Table I.

Table II gives the incidence of ovarian tumours in mice showing no oestrogen activity immediately prior to death and those showing some activity irrespective of its duration. The comparison indicates a high degree of correlation between oestrogen activity and the presence of tumours.

*Transplantability of the tumours.*

Four of the larger tumours arising in the pure IF mice were transplanted subcutaneously into other mice of the same strain. Two did not grow but the

TABLE II.—*Relation between Presence of Ovarian Tumours and Oestrogen Activity.*

| | Oestrogen activity. | No oestrogen activity. | Totals. |
|---|---|---|---|
| Ovarian tumours present | 39 | 9 | 48 |
| Ovarian tumours absent | 9 | 24 | 33 |
| | 48 | 33 | 81 |

$\chi^2 = 23{\cdot}6 \quad P < 0{\cdot}001$.

other 2 did. They were carried through another transplant successfully, but failed to grow after the third subcutaneous transplant. These tumours showed a preference for growth in male mice (Fig. 16).

Two tumours arising in IF × A hybrids were transplanted into similar hybrids of both sexes, but neither of them grew.

One tumour arising in an IF × C57 hybrid was transplanted into similar hybrids of both sexes and into pure IF mice, both males and castrated females. It grew successfully in hybrids of either sex and in one castrated female IF mouse. The second transplant failed

In cases where the transplant was successful, the tumour took about two months to grow to a size of about 1 cm. diameter. The successful transplants included tumours of both the pseudofollicular and cribriform types. In one example, where the original tumour showed only cribriform structure, the first generation transplant showed pseudofollicular structure.

There appeared to be no correlation between the transplantability of the tumours and their oestrogenic activity.

*Incidence of mammary carcinoma.*

Mammary carcinoma was induced during the course of the experiments in 65 of the mice, including the pure IF strain animals and both types of hybrid. All the animals may be presumed to have been free from the Bittner agent, and the histological structure of the mammary tumours was that of the chemically induced rather than the agent type (Orr, 1951). It is rather surprising that the incidence of mammary tumours does not significantly differ between mice with and without ovarian tumours, as is shown by a $\chi^2$ test on the data of Table III. If a comparison is made on the basis of the presence or absence of oestrogenic activity as determined by vaginal smears, a similar result is obtained (Table IV). On a quantitative basis, the number of breast tumours in individual mice bearing ovarian tumours varied between 0 and 5 with, an average of 1·33 ; the corresponding range in mice without ovarian tumours is 0 to 5, with an average of 1·48. It would therefore appear that the presence of detectable oestrogenic function is not necessary for the induction of breast carcinoma with DMB. It is known that castration will protect IF mice from the chemical induction of breast cancer with methylcholanthrene and will prevent the appearence of breast cancer in other strains carrying the Bittner agent. Corresponding data for DMB are not available.

### DISCUSSION.

*Chemical induction of ovarian tumours.*

Many papers have been published on the induction of ovarian tumours in rats and mice by exposure to a sterilising dose of X-rays, or by grafting a piece of ovary

TABLE III.—*Relative Incidence of Mammary and Ovarian Tumours in* 88 *IF, IF × A and IF × C57 Mice.*

| | Mammary tumours present. | Mammary tumours absent. | Totals. |
|---|---|---|---|
| Ovarian tumours present | 38 | 15 | 53 |
| Ovarian tumours absent | 27 | 8 | 35 |
| | 65 | 23 | 88 |

$\chi^2 = 0{\cdot}32$. $P > 0{\cdot}5$.

TABLE IV.—*Relation Between Presence of Breast Tumours and Oestrogen Activity.*

| | Oestrogen activity. | No oestrogen activity. | Totals. |
|---|---|---|---|
| Breast tumours present | 36 | 25 | 61 |
| Breast tumours absent | 12 | 8 | 20 |
| | 48 | 33 | 81 |

$\chi^2$ approximately 2. $P > 0{\cdot}10$.

to a site, such as the spleen, which is drained through the liver. The latter method is often accompanied by parabiosis of the grafted animal to a castrated animal. There are reports of experiments in which administration of a carcinogenic substance appeared to increase the rate of development of ovarian tumours by one of the above methods, but the chemicals alone were ineffective in producing tumours. Furth and Boon (1947) showed that the time of appearance of tumours in X-rayed mice was reduced from 9 months to 5 months by painting with methylcholanthrene. In 1951, Bielschowsky and Hall found that ovarian tumours could be produced in only 15 weeks in intact female mice treated with acetylaminofluorene and parabiosed to gonadectomised litter-mates, while in intact parabionts without acetylaminofluorene no tumours were found (though a suspicion of one was found in a pair surviving 42 weeks).

Engelbreth-Holm and Lefevre (1941) refer to ovarian tumours occurring in one out of 44 dilute brown and one out of 20 Aka mice treated with DMB. As far as we are aware, the results reported here are the first to indicate that tumours of the ovary can be induced in significant numbers by treatment of an animal with a chemical compound alone.

*X-ray induction of ovarian tumours.*

Changes in the ovaries of adult, non-parous female mice after irradiation were first described by Brambell and Parkes (1927). They found that the oöcytes degenerated and disappeared after 5 weeks, and no new follicles were formed. The granulosa and theca interna cells of some of the large follicles divided to fill the follicle cavity and then the whole structure became merged with surrounding tissue to form a mass of large vacuolated cells. A few follicles became cystic. Corpora lutea were found in all the animals. These showed signs of retrogression with shrinkage, vacuolation and fusion of the luteal cells.

Later changes leading to the development of ovarian tumours were noted by Furth and Butterworth (1936), Butterworth (1937) and Traut and Butterworth

(1937). They found that after about 150 days of degeneration, the germinal epithelium proliferated and invaginated to form a sort of adenoma with no hormonal activity by about 300 to 400 days. Granulosa-cell tumours were considerd to arise from surviving granulosa cells in a partially degenerated follicle. Luteomas occurring in some of the mice were believed to have originated as granulosa-cell tumours. Both these latter types of tumour produced oestrogen. Geist, Gaines and Pollack (1939) produced similar types of tumour with similar oestrogenic properties, but they thought that the tumours arose from a single-stem cell in the ovarian parenchyma. The descriptions and photographs of granulosa-celled tumours of all these authors resemble ours, but unlike most of them we have not satisfied ourselves that we have produced any tumour which could be described as a luteoma.

*Ovarian tumours in intrasplenic grafts.*

In 1944 Biskind and Biskind found that implantation of the ovaries of a rat into its spleen was followed by the development of tumour-like masses of granulosa cells about 300 days later. The same result was obtained by the intrasplenic grafting of a single ovary if the other were removed. In 1948 they found that if one ovary were left *in situ* the grafted ovary atrophied, whilst the intact one underwent compensatory hyperplasia. If the intact ovary were removed later the grafted one subsequently enlarged and appeared to be luteinised. No tumours occurred in ovaries grafted to, or adherent to sites not drained through the liver.

In the histogenesis of tumours formed from intra-splenic grafts of ovary in a castrate rat or mouse there is first inflammation. Then a few follicles develop and luteinise, but do not involute. In a few months the graft is a large mass of corpora lutea with a few scattered follicles. A nest of luteal cells then begins to grow and push aside the corpora lutea, and no more follicles form. In the resulting luteoma further changes sometimes occur. After 10 months or so nests of small cells appear and grow to form granulosa-cell tumours. Oestrus vaginal smears were usually, but not always, associated with granulosa-cell tumours (Biskind and Biskind, 1949).

It would therefore appear that in all the known methods of inducing ovarian tumours there is the common factor of an extensive reduction or disappearance of follicular tissue prior to the emergence of tumours. This is particularly evident in the case of our DMB experiments, where usually no viable follicles were present apart from the neoplastic pseudofollicles. In a few cases a very small number of abnormal follicles was present ; these tended to be grouped together in one small area of the atrophied ovary, were always of the anovular type, and raised the difficulty in interpretation of whether they were to be regarded as defective surviving follicles or incipient tumours. Histological survey of our material as a whole strongly suggests that disappearance or atresia of the normal follicles preceded rather than followed the development of tumours.

*Hormonal theory of ovarian tumour induction.*

As a result of their experiments in 1944 Biskind and Biskind put forward the following hypothesis of hormonal induction of these tumours. The transplantation of the ovaries to the spleen resulted in drainage of the oestrogens produced by them into the liver, where they were destroyed. The consequent lack of circulating oestrogen resulted in an increased discharge of gonadotrophic hormones from the pituitary, which in turn enhanced the growth of granulosa cells.

This theory has received much support, and Li and Gardner (1949) showed that no tumours developed in castrates with intra-splenic grafts of ovary if they were receiving oestradiol or testosterone, but progesterone did not inhibit tumour production. Intact ovarian function has also been shown to inhibit the growth of tumours in irradiated ovaries (Kaplan, 1950). Miller and Pfeiffer (1950) demonstrated increased gonadotrophin production in castrated mice with intra-splenic ovarian grafts by parabiosis experiments. These results have been expanded by Mühlbock (1951*a*).

The latent period of induction of the ovarian tumours is similar with X-rays and intrasplenic ovarian grafts. It is of the order of 9 or 10 months, but this period can be shortened in a way designed to destroy the circulating oestrogen more efficiently. Mühlbock (1953) has shown that the time of induction was decreased by parabiosing the X-rayed or grafted mouse to one or more castrated mice. The induction time was reduced in some cases to $3\frac{1}{2}$ months when two castrates were used.

It is difficult to conceive what is occurring in the mice treated with dimethylbenzanthracene. One could imagine that the pituitary mechanism may be involved in those mice whose history of oestrogen activity follows course (ii)—where oestrogen activity gradually disappeared altogether for a long period and suddenly reappeared, usually on the development of a small nodule of granulosa cell tumour. The long period of anoestrus would cause an increase in the gondadotrophic activity of the pituitary, which would in turn stimulate surviving granulosa cells. But what of the mice whose history followed course (iii)—where the oestrus phases gradually became longer and eventually permanent? In these mice there should be a corresponding decrease of pituitary gonadotrophins, yet many tumours arose in mice of this type.

*Transplantability of the ovarian tumours.*

Furth (1946) found that 13 of 21 attempts to transplant X-ray-induced ovarian tumours in mice succeeded, of which 11 were carried serially. Continued oestrus occurred in castrated or intact females bearing the tumours, and in the males the testes and seminal vesicles atrophied. Changes were also seen in the thymus of some of the mice, and there was frequent cavernous dilatation of liver, spleen and adrenal sinusoids. There were metastases to the liver and lung.

Tumours induced in intra-splenic grafts appear to be less malignant (Furth and Sobel, 1947) and may not be neoplastic in the accepted sense. Apart from 2 tumours which became neoplastic, subcutaneous fragments of the induced tumours failed to grow. Seven out of 44 would only grow in the spleens of castrates, but subcutaneous grafts in castrates or splenic grafts in non-castrates never grew. An intra-splenic ovarian tumour induced by Li (1948) in a castrate male was transplantable into castrate mice.

In our attempts to transplant tumours induced with dimethylbenzanthracene we were successful in 3 out of 7 cases. The transplants grew subcutaneously and 2 of them appeared to prefer to grow in males. We have not succeeded in growing them for more than two passages so far, and have not studied the hormonal aspects of the problem yet, but it can be stated that some of them will take in intact females.

*Mammary tumours.*

It has been shown that there is no significant correlation between the occurrence of mammary tumours and the presence of either ovarian tumours or hormonal activity. The IF strain and hybrids are particularly susceptible to the chemical induction of breast cancer apart from the Bittner agent, and up to the present we have been unable to make a similar comparison in other strains. In strains we have used which carry the Bittner agent the mammary tumours tended to appear very early, necessitating the killing of the mice before fully adequate time had elapsed to obtain reliable evidence on the ovarian reaction. Furth and Butterworth (1936) induced ovarian tumours with X-rays in three different strains of mice and found that the strain producing the largest number of ovarian tumours also had the highest breast tumour incidence. They do not give information on the degree of coincidence of breast and ovarian tumours in individual animals within this strain, and it should be remembered that their work preceded the discovery of the Bittner agent. Mülbock (1951*b*) X-rayed two strains of mice, dilute brown freed from the Bittner agent by Caesarean section and fostering, and C57 black (low breast-cancer and free from agent). He also used $F_1$ hybrids between the two strains. Granulosa-cell tumours occurred in the hybrids and in the dilute brown strain, but not in C57 blacks. It would appear however, that the agent-freed dilute brown mice no longer yielded mammary cancer, as the only change described in the breast is acinar hyperplasia indicating oestrogenic activity by the ovarian tumours.

*Spontaneous ovarian tumours in mice.*

We have never encountered spontaneous tumours of the ovary in any of the three strains employed in these experiments. Strong, Gardner and Hill (1937) found a granulosa-cell carcinoma in a non-irradiated EBA mouse which was transplantable into 32/99 mice over 7 transplants. The tumours caused continued oestrus from the 51st day in transplanted females. The mammary glands of males with grafts hypertrophied. Strong, Hill, Pfeiffer and Gardner (1938) report another ovarian tumour in a mouse which was transplantable into intact males 100 per cent, castrated males 45 per cent and less than 5 per cent in females, indicating that this tumour required androgenic support.

### SUMMARY.

Ovarian tumours have been found in 53 out of 88 virgin female mice treated at fortnightly intervals with an oily solution of 9 : 10-dimethyl-1 : 2-benzanthracene. The incidence does not significantly differ as between pure-strain Bonser IF mice and first generation hybrids derived from crossing IF females with Strong A or C57 Black males.

All the tumours appeared to be of granulosa-celled origin. Luteomata did not occur, although there was extensive luteinisation of the non-neoplastic ovaries.

Vaginal smears demonstrated some association between the presence of tumours and oestrogenic activity.

Some of the ovarian tumours are transplantable into intact females as well as males and castrated females.

The treatment also leads to mammary carcinoma, but its incidence is of the same order in mice without ovarian tumours as when they are present.

The results are compared with previous methods of inducing ovarian tumours by X-radiation or intrasplenic grafting.

This work was supported by the Birmingham Branch of the British Empire Cancer Campaign.

REFERENCES.

BIELSCHOWSKY, F. AND HALL, W. H.—(1951) *Brit. J. Cancer*, **5**, 331.
BISKIND, G. R. AND BISKIND, M. S.—(1948) *Science*, **108**, 137. (1949) *Amer. J. clin. Path.*, **19**, 501.—(1944) *Proc. Soc. exp. Biol., N.Y.*, **55**, 176.
BRAMBELL, F. W. R. AND PARKES, A. S.—(1927) *Proc. Roy. Soc.* B., **101**, 316.
BUTTERWORTH, J. S.—(1937) *Amer. J. Cancer*, **28**, 85.
Committee on Standardised Nomenclature for Inbred Strains of Mice (1952), *Cancer Res.*, **12**, 602.
ENGELBRETH-HOLM, J. AND LEFEVRE, H.—(1941)—*Ibid*, **1**, 102.
FURTH, J.—(1946) *Proc. Soc. exp. Biol. N.Y.*, **61**, 212.
*Idem* AND BOON, M. C.—(1947) *Cancer Res.*, **7**, 241.
*Idem* AND BUTTERWORTH, J. S.—(1936) *Amer. J. Cancer*, **28**, 66.
*Idem* AND SOBEL, H. (1947) *J. nat. Cancer Inst.*, **8**, 7.
GEIST, S. H., GAINES, J. A. AND POLLACK, A. D.—(1939) *Amer. J. Obstet. Gynec.*, **38**, 786.
KAPLAN, H. S.—(1950) *J. nat. Cancer Inst.*, **11**, 125.
LI, M. H. —(1948) *Amer. J. Obstet. Gynec.*, **55**, 316.
*Idem* AND GARDNER, W. U.—(1949) *Cancer Res.*, **9**, 35.
MARCHANT, JUNE, ORR, J. W. AND WOODHOUSE, D. L.—(1954) *Nature*, **173**, 307.
MILLER, O. J. AND PFEIFFER, C. A. (1950) *Proc. Soc. exp. Biol. N.Y.*, **75**, 178.
MÜHLBOCK, O.—(1951*a*) *Ned. Tijdschr. Geneesk.*, **95**, 3672.—(1951*b*) *Ibid.*, **95**, 915.—(1953) *Acta Endocr., Copenhagen*, **12**, 105.
ORR, J. W.—(1943) *J. Path. Bact.*, **55**, 483.—(1946) *Ibid.*, **58**, 589.—(1951) *Acta Un. Int. Cancr.*, **7**, 294.
SHORR, E.—(1941) *Science*, **94**, 545.
STRONG, L. C., GARDNER, W. U. AND HILL, R. T.—(1937) *Endocrinology*, **21**, 268.
*Idem* HILL, R. T., PFEIFFER, C. A. AND GARDNER, W. U.—(1938) *Genetics*, **23**, 585.
TRAUT, H. F. AND BUTTERWORTH, J. S.—(1937) *Amer. J. Obstet. Gynec.*, **34**, 987.